# Low Body Weight Increases the Risk of Ischemic Stroke and Major Bleeding in Atrial Fibrillation: The COOL-AF Registry

**DOI:** 10.3390/jcm9092713

**Published:** 2020-08-22

**Authors:** Rungroj Krittayaphong, Ply Chichareon, Chulalak Komoltri, Sakaorat Kornbongkotmas, Ahthit Yindeengam, Gregory Y. H. Lip

**Affiliations:** 1 Faculty of Medicine Siriraj Hospital, Mahidol University, Bangkok 10700, Thailand; chulaluk.kom@mahidol.ac.th (C.K.); porpor_2@hotmail.com (A.Y.); 2 Cardiology unit, Department of Internal Medicine, Faculty of Medicine, Prince of Songkla University, Songkla 90110, Thailand; plyc83@yahoo.com; 3 Queen Savang Vadhana Memorial Hospital, Chonburi 20110, Thailand; hembae174@gmail.com; 4 Liverpool Centre for Cardiovascular Science, University of Liverpool and Liverpool Heart & Chest Hospital, Liverpool L14 3PE, UK; Gregory.Lip@liverpool.ac.uk; 5 Aalborg Thrombosis Research Unit, Department of Clinical Medicine, Aalborg University, DK-9100 Aalborg, Denmark

**Keywords:** low body weight, ischemic stroke, major bleeding, Thai patients, non-valvular atrial fibrillation, COOL-AF Registry

## Abstract

We aimed to determine if low body weight (LBW) status (<50 kg) is independently associated with increased risk of ischemic stroke and bleeding in Thai patients with non-valvular atrial fibrillation (NVAF). (1) Background: It has been unclear whether LBW influence clinical outcome of patients with NVAF. (2) Methods: This prospective multicenter cohort study included patients enrolled in the COOL-AF Registry. The following data were collected: demographic data, medical history, risk factors and comorbid conditions, laboratory and investigation data, and medications. Follow-up data were collected every 6 months. Clinical events during follow-up were confirmed by the adjudication committee. (3) Results: A total of 3367 patients were enrolled. The mean age was 67.2 ± 11.2 years. LBW was present in 338 patients (11.3%). Anticoagulant and antiplatelet was prescribed in 75.3% and 26.2% of patients, respectively. Ischemic stroke, major bleeding, intracerebral hemorrhage (ICH), and death occurred during follow-up in 2.9%, 4.4%, 1.4%, and 7.7% of patients, respectively, during 25.7 months follow-up. LBW was an independent predictor of ischemic stroke, major bleeding, ICH, and death, with a hazard ratio of 2.40, 1.79, 2.37, and 2.65, respectively. (4) Conclusions: LBW was independently associated with increased risk of adverse outcomes in Thai patients with NVAF. This should be carefully considered when balancing the risks and benefits of stroke prevention among patients with different body weights.

## 1. Introduction

Non-valvular atrial fibrillation (NVAF) can lead to the development of thromboembolism and subsequent ischemic stroke [1]. Practice guidelines for oral anticoagulation (OAC) management in patients with NVAF suggest that patients with a CHA2DS2-VASc score of 1 should be considered for OAC treatment [2,3,4].

The Asian population is at increased risk for developing ischemic stroke [5] and intracerebral hemorrhage [6,7]. Even patients with a CHA2DS2-VASc score of 0 may have an ischaemic stroke risk of 1.15% per year [8], leading to a modified CHA2DS2-VASc score being proposed for Asian patients [9]. In Asian patients, body weight may influence the risk of stroke [10], which is particularly relevant since Asian peoples are often of smaller stature and lower weight compared to white Europeans. Of note, being of low body weight (LBW) might increase stroke risk in both white European [11] and Asian populations [12]. LBW also increases the risk of bleeding, especially among those who are on OAC [12]. In those taking vitamin K antagonist (e.g., warfarin), the dose of warfarin to achieve the target international normalized ratio (INR) may be lower in LBW patients due to a smaller volume of drug distribution; however, the likelihood of the development of complications after drug dose adjustment was reported to be higher in LBW patients [13]. Dose adjustment of certain non-vitamin K antagonist oral anticoagulants (NOACs) is recommended in patients with a lower body weight [14].

The COOL-AF registry is a prospective nationwide registry of newly diagnosed patients with NVAF, conducted in multiple centers in Thailand, as previously described [15]. Given that many Thai patients are of LBW (typical of many Asian peoples), the aim of this study was to determine if LBW status (<50 kg) was independently associated with increased risk of ischemic stroke and bleeding in Thai patients with NVAF.

## 2. Methods

### 2.1. Study Population

In COOL-AF, we prospectively and consecutively enrolled patients aged 18 years or older with definitively diagnosed NVAF by 24-lead electrocardiograph (ECG) or Holter monitoring at one of 27 large hospitals located throughout Thailand. A list of investigators is shown in Supplemental materials. Patients with any of the following were excluded: mechanical heart valves; rheumatic valve disease; hematologic conditions that increase the risk of bleeding; NVAF from transient reversible causes; life expectancy less than 3 years; unable to attend follow-up visits; refusal to participate; ischemic stroke within the preceding 3 months; pregnant at the time of the eligibility assessment; and participation in a clinical trial at the time of the eligibility assessment. The Institutional Review Board (IRB) of each participating hospital approved the study protocol and all enrolled patients approved their participation by providing written informed consent.

### 2.2. Study Protocol and Data Collection

Each patient was fully informed about the purpose of and the details relating to the study. Patient data were collected from a review of patient medical records and from the patient interview. Required data were written in the case record form (CRF). Data from the CRF were then transferred into the web-based data collection and management system and the hard copy of the CRF was sent to the central data management unit. Upon receiving the hard copy of the CRF, the study team at the data management unit cross-checked the data on the form with the data entered into the web-based system. Discrepancies of any kind were addressed to the study team at that study site. When the patient data for each patient was determined to be correct and complete, the data were locked and could not be changed. Follow-up data were collected and reviewed in a similar manner. Data were collected every 6 months until the last visit at 3 years after enrollment.

The following data were collected at the baseline visit: demographic data; weight and height; vital signs; type, duration and symptoms of NVAF; risk factors, such as diabetes and hypertension; comorbidities, such as coronary artery disease (CAD), heart failure (HF), or stroke; and all current medications for medical illnesses and prescribed antithrombotic agents, including OAC and antiplatelet drugs. Each component of CHA2DS2-VASc and HAS-BLED score was also collected. Clinical events and changes in risk factors and/or medications were collected at each follow-up visit.

### 2.3. Outcome Measurement

The evaluated clinical outcomes during follow-up were ischemic stroke or transient ischemic attack (TIA), major bleeding, intracerebral hemorrhage, and death. The research team at each study site was instructed to upload the source document into the web-based system to confirm the evidence of each event. All events were reviewed and validated by the study team at the data management unit. After validation, all data were sent to the adjudication committee for final evaluation and confirmation. In cases with inconclusive evidence or further questions, additional data or an explanation was requested from the study site. Ischemic stroke was defined as a sudden-onset neurologic deficit that persisted for longer than 24 h. TIA was defined as a sudden-onset neurologic deficit that lasted not longer than 24 h. International Society of Thrombosis and Haemostasis (ISTH) criteria [16] was used to define major bleeding, including fatal bleeding, bleeding in critical organs, bleeding that results in a decrease in hemoglobin level of 20 g/L or more, or bleeding that requires a transfusion of 2 units of red cells or more.

### 2.4. Statistical Analysis

Descriptive statistics were used to summarize patient demographic and clinical characteristics. LBW was defined as a body weight less than 50 kg. Mean ± standard deviation was used to describe continuous data and number and percentage were used to present categorical data. Comparisons between patients with body weight <50 and ≥50 kg were performed using Student’s *t*-test for unpaired continuous data and chi-square test for categorical data. Comparisons of clinical outcomes between and among groups were made using the chi-square test. Associations between LBW and clinical outcomes over time were tested by the Cox proportional hazards model. Kaplan-Meier estimate with log-rank test was performed to determine significant differences among groups. The results of that analysis are reported as hazard ratio (HR) and 95% confidence interval (CI) and are graphically displayed as the cumulative event rate compared between groups. Adjusted HR and 95% CI was also reported as Forest plot after adjustment for age, sex, comorbid conditions (CAD, HF, smoking status, diabetes, hypercholesterolemia, hypertension, history of ischemic stroke, history of major bleeding, renal replacement therapy, cardiac implantable electronic devices) and antithrombotic medications (OAC and antiplatelet). Generalized Estimating Equation (GEE) with exchangeable correlation structure was used to analyse the effect of LBW on clinical outcomes with the adjustment of time varying covariates. Cox regression models with restricted cubic splines were used to determine the association between body weight as a continuous variable and the rates of clinical outcomes adjusted for age, sex, and comorbid conditions. Net clinical benefit (NCB) of OAC versus no OAC was calculated using the formula: (ischemic stroke/TIA rate off OAC-ischemic stroke/TIA rate on OAC)-1.5 (ICH rate on OAC-ICH rate off OAC) [17]. NCB of NOAC versus warfarin was calculated in a similar manner. A *p*-value of <0.05 was considered statistically significant. All statistical analyses were performed using SPSS Statistics version 23 (SPSS, Inc., Chicago, IL, USA, www.ibm.com/analytics/spss-statistics-software), R version 3.6.3 (www.r-project.org), and SAS studio (SAS Institute Inc., Cary, NC, USA, www.sas.com/en_th/contact.html).

## 3. Results

We enrolled a total of 3367 patients (mean age 67.2 ± 11.2 years; 58.5% male) in this study. The average CHA2DS2-VASc and HAS-BLED scores were 3.05 ± 1.68 and 1.53 ± 1.01, respectively. The distributions of body weight among male patients, female patients, and those aged ≥65 years and age < 65 years are shown in Figure S1 in Supplemental Material. The average body weight was 66.1 ± 14.5 kg; of these, 381 (11.3%) were classified as having LBW status. Table 1 shows baseline demographic and clinical characteristics compared between patients with body weight <50 and ≥50 kg. Patients with LBW were significantly older; more likely to be female; less likely to have hypertension, diabetes, dyslipidemia, and be a current smoker; had higher CHA2DS2-VASc and HAS-BLED scores; and were less likely to use antiplatelet drugs.

### 3.1. LBW and Outcomes

The average follow-up duration was 25.7 ± 10.6 months. Among the main clinical outcomes, ischemic stroke/TIA, major bleeding, ICH, and death occurred in 99 (2.9%), 148 (4.4%), 48 (1.4%), and 260 (7.7%) patients, respectively.

**Figure 1:**
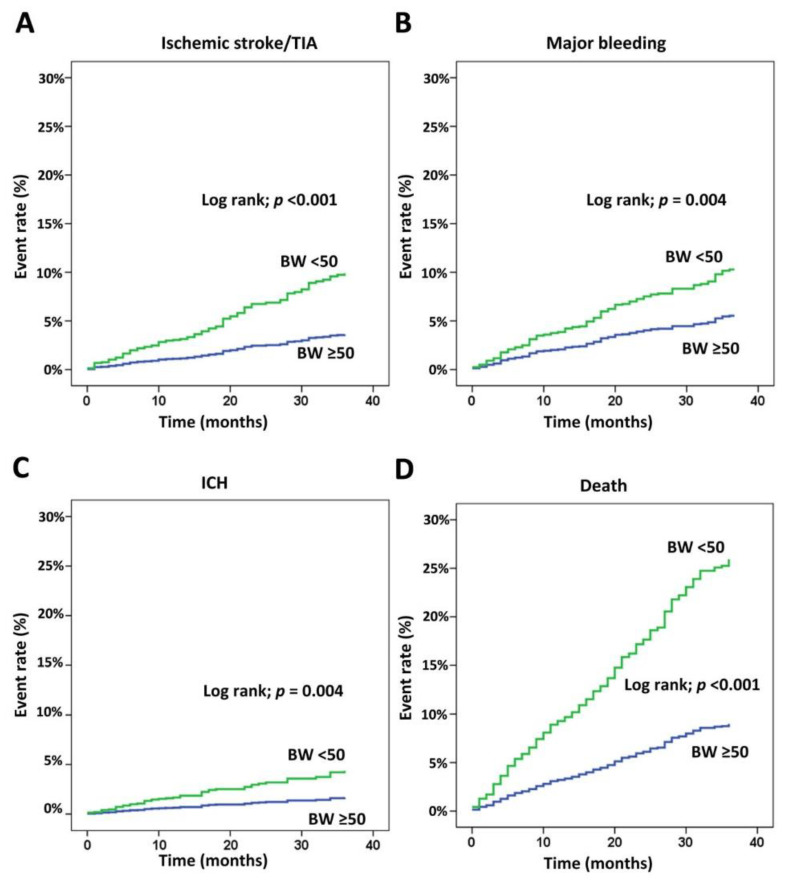
Cumulative event rate of ischemic stroke (IS)/transient ischemic attack (TIA) (**A**), major bleeding (**B**), intracerebral hemorrhage (ICH) (**C**), and death (**D**) compared between patients with body weight (BW) <50 and ≥50 kg. A *p*-value < 0.05 indicates statistical significance. Kaplan-Meier graph with log-rank test was used for analysis. BW, body weight; TIA, transient ischemic attack.

LBW increased the risk of ischemic stroke, major bleeding, ICH, and death with a hazard ratio (HR; 95% confidence interval (CI)) of 2.40 (1.51–3.83), 1.79 (1.18–2.72), 2.37 (1.21–4.65), and 2.65 (1.99–3.53), respectively. Rate of ischemic stroke/TIA, major bleeding, ICH, and death were 3.20, 3.78, 1.53, and 8.76 per 100 persons-year for low BW and 1.19, 1.89, 0.58, and 3.08 per 100 persons-year for non-LBW, respectively. The cumulative event rate is graphically shown for all four clinical outcomes compared between patients with body weight <50 and ≥50 kg in Figure 1. These graphs show a statistically significant difference between body weight groups for all four clinical outcomes and the difference in the number of events continued to widen over the follow-up duration for all 4 clinical event subgroups.

Regarding the cause of death, LBW had a higher rate of cardiovascular death (6.3% vs. 2.1%, *p* < 0.001). There was no significant difference in the rate of infection/sepsis and cancer as a cause of death between LBW and non-LBW group (2.9% vs. 1.8%, *p* = 0.147, for infection/sepsis, and 0.5% vs. 0.4%, *p* = 0.683, for cancer).

### 3.2. Subgroup Analyses

**Figure 2:**
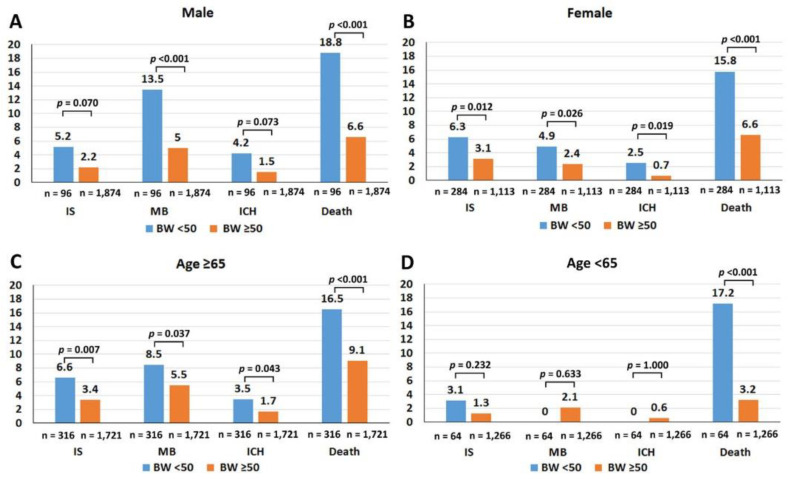
Rates of ischemic stroke (IS)/transient ischemic attack (TIA), major bleeding (MB), intracerebral hemorrhage (ICH), and death compared between body weight (BW) < 50 and ≥50 kg among male patients (**A**), female patients (**B**), patients aged ≥65 years (**C**), and patients aged <65 years (**D**). The chi-square test was used for analysis. A *p*-value < 0.05 indicates statistical significance.

Figure 2 shows the rates of ischemic stroke/transient ischemic attack, major bleeding, intracerebral hemorrhage, and death compared between body weight <50 and ≥50 kg among male patients, female patients, patients aged ≥65 years, and patients aged <65 years. Patients with LBW were at significantly increased risk for all clinical outcomes among all patients as well as in each subgroup, except among those aged <65 years. The small number of patients in the latter age subgroup may have limited any statistically significant association. Females had a significantly higher rate of ischemic stroke than males, whereas males had a significantly higher rate of major bleeding compared to females. The older adult population had a significantly higher rate of all clinical events compared to the younger adult group.

The rate of clinical outcomes among patients with body weight < 50 and ≥50 kg is shown in Table 2. Ischemic stroke, major bleeding, and ICH were all significantly increased when body weight was below 50 kg.

### 3.3. Multivariate Analysis

**Figure 3:**
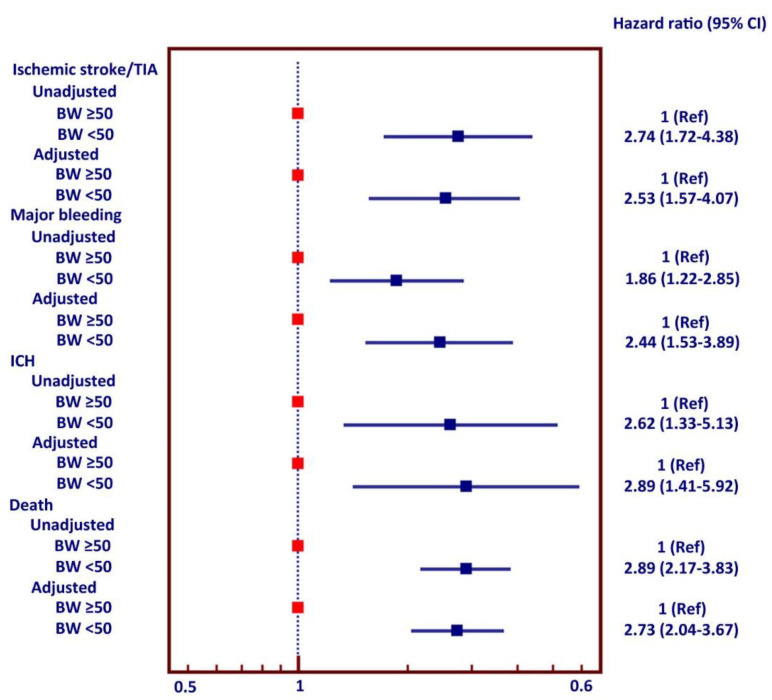
Forest plot shows unadjusted and adjusted hazard ratio of ischemic stroke/transient ischemic attack (TIA), major bleeding, intracerebral hemorrhage (ICH), and death in patients with low bodyweight and non-low body weight. The Cox proportional hazards model was used for analysis.

Associations between adverse clinical outcomes and low body weight alone and low body weight adjusted for different factors are shown as a Forest plot in Figure 3. LBW was independently associated with increased risk of all main clinical outcomes, including ischemic stroke, major bleeding, ICH, and death. LBW remained independently associated with all four of these clinical outcomes after adjustment for all three different sets of potential confounders.

### 3.4. Body Weight as a Continuous Variable

**Figure 4:**
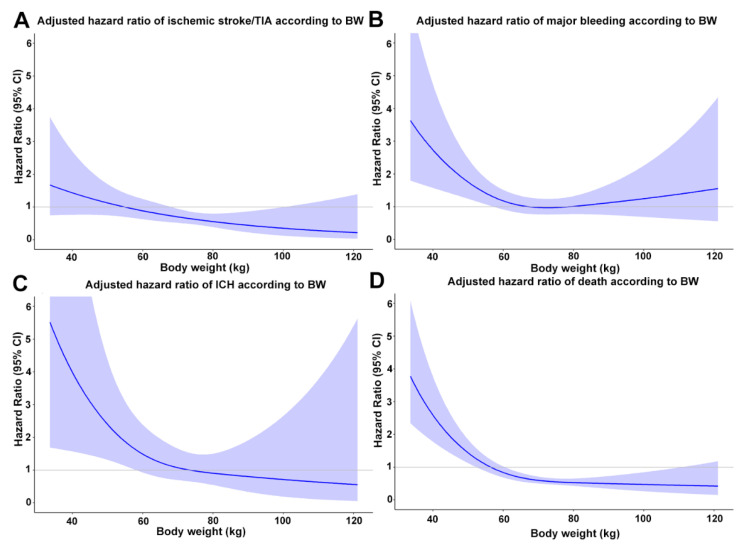
Restricted cubic splines graph showing Hazard ratio (95%CI) of ischemic stroke/transient ischemic attack (TIA) (**A**), major bleeding (**B**), intracerebral hemorrhage (ICH) (**C**), and death (**D**) in patients with different body weight adjusted for age, sex, and comorbid conditions (coronary artery disease, heart failure, smoking status, diabetes, hypercholesterolemia, hypertension, history of ischemic stroke, history of major bleeding, renal replacement therapy, cardiac implantable electronic devices). Cox regression models with restricted cubic splines were used for analysis. CI, confidence interval.

Cox models with restricted cubic splines by treating body weight as continuous data demonstrated that LBW increased the risk of ischemic stroke/TIA, major bleeding, ICH, and death (Figure 4). The risk of all clinical events increased when body weight was below 60 kg and markedly increased when the body weight was below 50 kg.

### 3.5. Sensitivity Analysis

We performed additional analysis for low body mass index (BMI) (<18.5 kg/m^2^) which is considered underweight by Asia-Pacific criteria [18] as compared to other BMI groups for measurement outcomes. We demonstrated that the underweight group had a higher rate of ischemic stroke/TIA, major bleeding, ICH, and death compared to the non-underweight group, with *p* values of 0.003, 0.007, 0.024, and <0.001, respectively.

During the average follow-up duration of 25.7 ± 10.6 months, there were some changes in risk factors and medications that might have influence on the outcomes. The rate of new development of specific risk factors during follow-up were 4.75% for heart failure, 6.37% for hypertension, 8.94% for age ≥ 75 years, 3.63% for diabetes, 3.04% for prior stroke or TIA, 1.04% for vascular disease, and 8.63% for age 65–74 years. OAC and antiplatelet were introduced during follow-up in 30.65% and 5.31%, whereas the discontinuation rate was 0.47% and 6.69%, respectively. The Generalized Estimating Equation (GEE) with exchangeable correlation structure was used to analyse the effect of LBW on clinical outcomes, with the adjustment of time varying covariates. The results of the analysis demonstrated that LBW remained an independent predictor for ischemic stroke/TIA, major bleeding, and ICH with the adjusted Odds ratio of 2.18 (1.27–3.74), *p* = 0005; 2.07 (1.26–3.39), *p* = 0.004; and 3.15 (1.47–6.77), *p* = 0.003, respectively.

Although we tried to adjust for potential confounders, there might be a concern whether the effect of LBW on clinical outcomes is due to a difference in baseline characteristics between the two groups such as CHA2DS2-VASc score, gender, and age. We, therefore, selected only patients with CHA2DS2-VASc score ≥2 and ran the analysis for the effect of LBW on clinical outcomes. The results showed that LBW remained a significant predictor for clinical outcomes, with the adjusted HR for LBW for ischemic stroke/TIA, major bleeding, ICH, and death being 2.64 (1.63–4.27), *p* < 0.001; 2.47 (1.56–3.91), *p* < 0.001; 3.19 (1.55–6.58), *p* = 0.002; and 2.47 (1.83–3.35), *p* < 0.001, respectively.

Regarding the influence of gender and age on the results of LBW on clinical outcomes, we used an interaction test. The results demonstrated that there was no significant interaction of gender or age group on the effect of LBW on clinical outcomes. The interaction *p*-values of gender were 0.637, 0.406, 0.734, and 0.279 for ischemic stroke/TIA, major bleeding, ICH, and death, respectively. The interaction *p*-values of the elderly (≥65 years) and non-elderly (<65 years) were 0.337, 0.729, 0.257, and 0.297 for the respective outcomes. Figure S2 in Supplementary Materials shows a restricted cubic splines graph of LBW and ischemic stroke/TIA and major bleeding stratified by gender (A and B) and age group (C and D).

To determine whether the body weight cut off is different for males and females, we ran a restricted cubic splines graph of ischemic stroke/TIA and major bleeding stratified by gender (Figure S2A,B in Supplementary Material) using body weight as a continuous variable. We demonstrated that the adjusted hazard ratio of ischemic stroke/TIA started rising at the body weight below 60 kg in both genders and significantly increased when body weight was below 50 kg. For major bleeding, the adjusted hazard ratio for males started rising at body weight below 65 kg and significantly increased at body weight below 50 kg. For females, the adjusted hazard ratio was increased at body weight below 50 kg.

For concern whether the reference group should be relatively normal weight individuals, we performed additional analysis. Since our cut off for LBW was approximately 10 percentiles, we ran an analysis for the effect of LBW on clinical outcomes by excluding patients with body weight above the 90th percentile. The adjusted hazard ratios of LBW compared to the reference group were 2.40 (1.49–3.88), *p* < 0.001; 2.25 (1.42–3.55), *p* = 0.001; 2.85 (1.39–5.86), *p* = 0.004; and 2.74 (2.04–3.69), *p* < 0.001 for ischemic stroke/TIA, major bleeding, ICH, and death, respectively.

### 3.6. Effects of OAC on Clinical Outcomes

**Figure 5:**
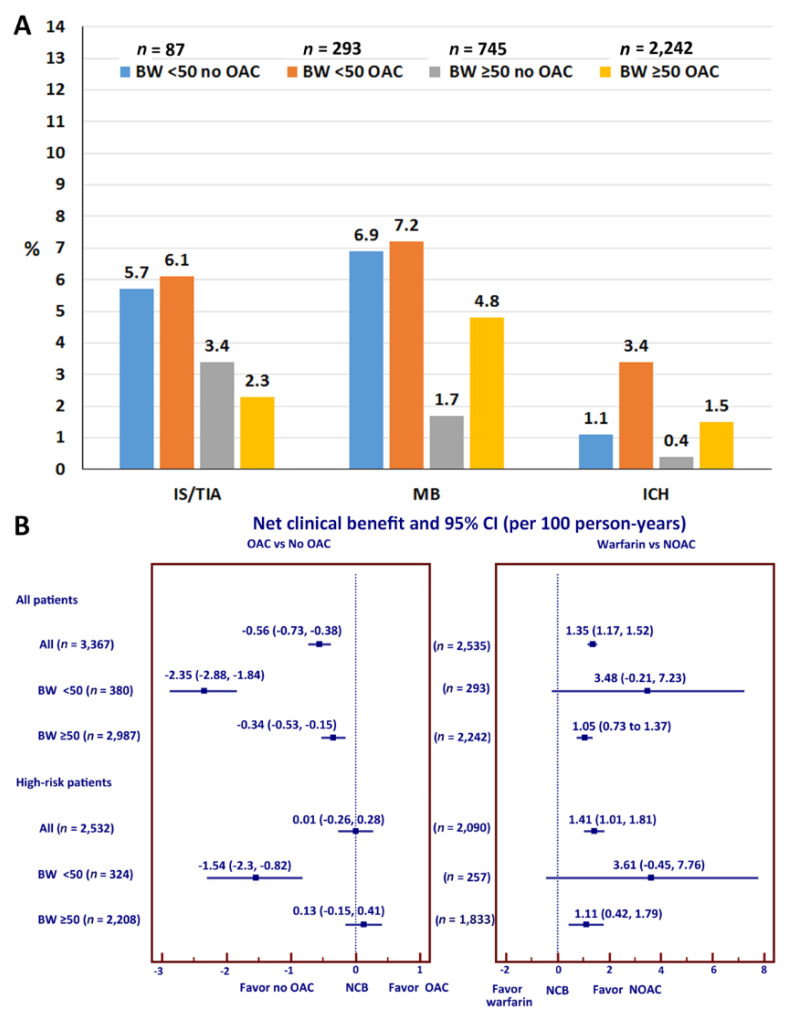
Oral anticoagulants and clinical outcomes. (**A**). Rates of ischemic stroke (IS)/transient ischemic attack (TIA), major bleeding (MB), and intracerebral hemorrhage (ICH) stratified by body weight (BW) and oral anticoagulation (OAC). The interaction *p*-values of OAC use for the effect of body weight on ischemic stroke/TIA, major bleeding, and ICH were 0.025, 0.871, 0.981, respectively. (**B**). Net clinical benefit (NCB) of OAC compared to no OAC (left) and warfarin compared to non-vitamin K antagonist oral anticoagulant (NOAC) (right). Forest plot and Cox proportional hazards model was used for analysis.

Additional analyses were performed to evaluate the effect of OAC use on the rate of clinical outcomes among patients with different body weights (Figure 5A). In patients with LBW, OAC use was not associated with lowered ischemic stroke/TIA but an increased risk of major bleeding. OAC use increased the risk of ICH greater than 3-fold compared to those not on OAC. The risk of ICH also increased approximately 3-fold in patients with body weight ≥ 50 kg compared to those not on OAC (3.4% vs. 1.1%). The risk of ICH in patients on OAC with body weight < 50 kg was more than two-fold compared to those with body weight ≥ 50 kg (3.4% vs. 1.5%). The interaction *p*-values of OAC use for the effect of body weight on ischemic stroke/TIA, major bleeding, ICH, and death were 0.025, 0.871, 0.981, and 0.954, respectively. For those who were on warfarin, average time in therapeutic range (TTR) was 53.5 ± 26.4%. TTR was 53.0 ± 26.2% in patients with low BW compared to 53.6 ± 26.4% for those without low BW (*p* = 0.722).

### 3.7. Net Clinical Benefit

NCB was calculated for all patients and for high-risk patients defined as men with CHA2DS2-VASc score more than 1 or women with CHA2DS2-VASc score more than 2. When weighting ICH as 1.5 as mentioned earlier, NCB (95% CI) of OAC compared to no OAC was −0.56 (−0.73 to −0.38) per 100 person-years, which means that OAC may have a higher risk than benefit. NCB was more negative (more risk than benefit) in patients with LBW (−2.35 (−2.88 to −1.84)) per 100 person-years] compared to non-LBW (−0.34 (−0.53 to −0.15)) per 100 person-years] group (Figure 5B). Warfarin was used in 2310 (91.1%) of patients who are on OAC. Our study had limited power to compare outcomes of warfarin and NOACs due to the small number of NOACs, especially in the LBW group. However, the results of NCB showed that NOACs had NCB of 1.35 (1.17 to 1.52) per 100 person-years compared to warfarin, which means that NOAC provided more benefit than risk compared to warfarin. NCB of NOACs compared to warfarin in LBW and non-LBW were 3.48 (−0.21 to 7.23) and 1.05 (0.73 to 1.37) per 100 person-years, respectively, which means that the benefit of NOACs over warfarin was more in the LBW group. Results of NCB in high-risk patients was in a similar direction for all patients.

## 4. Discussion

In this prospective nationwide cohort of Thai patients with atrial fibrillation (AF), we show that LBW was present in 11.3% and was an independent predictor of increased risk of ischemic stroke, major bleeding, ICH, and death irrespective of OAC therapy. This is the largest contemporary cohort of Thai patients with NVAF. 

Concerns whether LBW status increases the chance of developing a bleeding complication from OAC treatment has been reported [19]. A smaller body size means a smaller volume of distribution and thus, a regular dose of medication might increase the blood concentration of that medication to a level higher than would be achieved in someone with a normal body size [13]. This is particularly important for OAC medications, which may cause bleeding at inappropriately high blood concentrations. Certain OAC medications, such as apixaban and edoxaban, are recommended for consideration of a low-dose regimen in LBW patients [14].

Asians have a smaller average body size than Caucasians and there are concerns that Asian patients may be at higher risk for developing adverse effects from medications compared to larger body size counterparts [20]. For example, many anticoagulant drugs have a “Japanese dose” that is lower than the usual recommended dose [21]. Other studies have reported that the Asian population is more likely to have bleeding complications than white Europeans [7,22]. Indeed, warfarin-related ICH complications in NVAF patients are 4 times higher in Asian population than in Caucasian populations [7]. The recent NOAC trials have also showed that the Asian subgroup had higher rates of both major bleeding and ICH when on warfarin therapy [22]. This could be one of the reasons why a low proportion of the Asian population received OAC compared to Western populations [23] and even when they did receive warfarin, the proportion that had an INR within the target range was lower than that of patients in other countries [23].

The prevalence of NVAF in elderly Thai participants was 1.9% [24]. Only 40% of known AF subjects received warfarin while 60% received antiplatelets. The prevalence of underweight people among the Thai population was approximately 8.7% [25]. From Thailand national data on 522,699 patients with acute stroke, NVAF is one of the major factors leading to stroke [26].

Some previous studies reported that LBW increased the risk of adverse events in patients with NVAF [11,12]. Data from the Fushimi AF Registry showed that LBW significantly increased the risk of ischemic stroke among 2945 NVAF patients (HR: 2.19, 95% CI: 1.57–3.04), but the risk of bleeding was comparable between the LBW and normal body weight groups (HR: 1.05, 95% CI: 0.64–1.68). However, only about 50% of patients in the Fushimi AF Registry received OAC therapy [12]. In contrast, OAC was given to 75.3% of patients in our registry. The Fushimi AF Registry also showed the risk of death to be almost 3 times higher in the LBW group compared to the non-LBW group [12]. A study from the Korean National Health Insurance Database also found that patients with a body weight < 50 kg had a higher incidence of ischemic stroke, bleeding, and death compared to those with a body weight ranging from 50 to 60 kg [27]. These results are consistent with our findings (Table 2) that the risk of both ischemic stroke and bleeding was significantly increased when body weight was below 50 kg. It should be noted that 100% of patients in the Korean study were on OAC and NOAC was used in 65% of their patients. In contrast, NOAC was used in only 9% of patients in our study.

A systematic review and meta-analysis compared data from randomized clinical trials of NOACs in patients with NVAF or venous thromboembolism (VTE) and showed that LBW increased the risk of ischemic stroke, but the rate of major bleeding was comparable between the LBW and non-LBW groups [11]. That group also compared obese versus non-obese patients and found that obese patients had a lower rate of ischemic stroke compared to non-obese patients, but the rate of major bleeding was not significantly different between the two weight groups [11]. Those findings are consistent with our observations that the rate of ischemic stroke was lower in patients with body weight ≥ 60 kg compared to <60 kg and the rate markedly increased when body weight < 50 kg. However, their finding of Boonyawat et al. [11] of a comparable bleeding event rate between the LBW and non-LBW groups is in contrast to our results. One possible explanation could be the weight cutoff used in the included studies. More specifically, half of the studies they included used 50 kg as the cutoff and the other studies used 60 kg as the cutoff. Once again, we found the rates of both ischemic stroke and bleeding to be significantly increased when body weight was below 50 kg. The increased risk of major bleeding in the LBW group of our study cannot be explained by the rate of OAC use, there was no significant difference in the 2 groups (77.1% for LBW and 75.1% for non-LBW group, *p* = 0.384), and it cannot be explained by the rate of OAC plus antiplatelet, which was more common in the non-LBW group (4.2% for LBW and 9.8% for non-LBW group, *p* < 0.001). The use of NOAC may be the solution for ischemic stroke and major bleeding risk reduction. Our study also demonstrated that the NCB results favored NOAC over warfarin for both the LBW and non-LBW group. The NCB of NOAC in the LBW group was greater than the non-LBW group. Recent meta-analysis demonstrated that use of NOAC contributed to the reduction of both ischemic stroke and major bleeding across all groups of body weight [28]. The results of our study did not have enough power to analyze the effect of NOAC on clinical outcomes due to the limited number of patients using NOAC. However, data from our study showed that compared to warfarin, the rate of major bleeding was significantly lower with NOAC (5.4% for warfarin, 2.2% for NOAC, *p* = 0.040) and the rate of ischemic stroke/TIA was numerically lower for NOAC (2.9% for warfarin, 1.3% for NOAC, *p* = 0.180). A slightly higher time in the therapeutic range in the LBW group in our study should not explain the increased risk of adverse outcomes in the LBW group since the difference is small, the average time in the therapeutic range is similar, and the LBW group also had increased risk of ischemic stroke/TIA, not just major bleeding.

We use 50 kg as the cut off criteria for low body weight due to the following reasons: (1) weight is a simple measure and it is easier to use than BMI, which needs a calculation. For example, one of the dose reduction criteria of apixaban is body weight less than 60 kg (2) we use less than 50 kg to compare the results with previous publications [11,12]. We performed additional analysis comparing BMI using the cut off of 18.5 kg/m^2^, which is considered underweight by Asia-Pacific population criteria [18], and normal weight (18.5–22.9 kg/m^2^), overweight (23–24.9 kg/m^2^), and obesity (≥25 kg/m^2^). Patients in the underweight group had a higher rate of a composite of ischemic stroke/TIA, major bleeding, and death compared to the other three groups (23.7% vs. 17.2% vs. 10.3% vs. 9.3%, respectively). The rate of individual clinical outcomes was also higher in the underweight group defined by the BMI.

Several mechanisms for the linkage between LBW and increased risk of ischemic stroke and bleeding have been proposed. First, LBW may be associated with an altered pharmacokinetic effect of the anticoagulant, which increases bleeding risk [13]. Second, these increased risks could be related to genetic factors that are also associated with smaller body size [20]. Third, LBW patients may have a lower lean body mass, which was reported to be associated with a worse prognosis for cardiovascular outcome [29]. Fourth, obese patients tended to adhere to guideline recommended therapy more closely than their lower body weight counterparts [30]. Fifth, obese patients tend to have an increased level of cardioprotective adipokines [31,32] and LBW is associated with impaired endothelium-dependent vasodilatation [33]. Sixth, patients with LBW may have a different neuro-hormonal response to exercise [34]. In addition to LBW being shown to increase the risk of cardiovascular events in patients at high risk for atherothrombotic events [35], levels of inflammatory markers are also increased in patients with LBW [36,37]. Lastly, people from many Asian countries had a less favorable body composition and adipokines. Although they might have a lesser lean body mass, many of them had a greater visceral fat component, had lower adiponectin levels, and higher resistin levels, creating a metabolically abnormal normal weight phenotype with unfavorable metabolic health, even at normal weight or underweight [38].

### Limitations

This study has some limitations. First, the results of this study may not be generalizable to Thai patients in other care settings or to patients that are not Thai. The patients in this registry were mainly enrolled from large hospitals and most received their care from cardiologists. In contrast, patients that receive care in less sophisticated settings are often treated by internists or general practitioners. Second, OAC in our study was mainly warfarin, which may limit generalizability to populations with a large proportion of NOAC usages. Besides, we did not have data on the dose of NOACs that might have an effect on the clinical outcomes. The strength of this study is its prospective design, the large size of the study population, and the fact that the data were collected from 27 large hospitals located all across Thailand.

## 5. Conclusions

LBW was independently associated with increased risk of ischemic stroke, major bleeding, ICH, and death in Thai patients with NVAF. OAC may not have benefit in a LBW group. This should be carefully considered when balancing the risks and benefits of stroke prevention among patients with different body weights.

## Figures and Tables

**Table 1:** Baseline characteristics compared between patients with body weight < 50 and ≥50 kg.

Characteristics	All (*n* = 3367)	BW < 50 (*n* = 380)	BW ≥ 50 (*n* = 2987)	*p*
Age (years)	67.2 ± 11.2	74.1 ± 10.2	66.4 ± 11.1	<0.001
Female	1397 (41.5%)	284 (74.7%)	1113 (37.3%)	<0.001
Time after NVAF diagnosis (years)	3.4 ± 4.3	3.2 ± 3.9	3.4 ± 4.4	0.362
Type of NVAF				0.070
- Paroxysmal	1131 (33.6%)	115 (30.3%)	1016 (34.0%)	
- Persistent	642 (19.1%)	64 (16.8%)	578 (19.4%)	
- Permanent	1594 (47.3%)	201 (52.9%)	1393 (46.6%)	
Symptomatic NVAF	2600 (77.2%)	292 (76.8%)	2308 (77.3%)	0.852
History of heart failure	897 (26.6%)	93 (24.5%)	804 (26.9%)	0.310
History of coronary artery disease	543 (16.1%)	44 (11.6%)	499 (16.7%)	0.010
CIED	329 (9.8%)	45 (11.8%)	284 (9.5%)	0.149
History of ischemic stroke/TIA	573 (17.0%)	72 (18.9%)	501 (16.8%)	<0.001
Hypertension	2299 (68.3%)	229 (60.3%)	2070 (69.3%)	<0.001
Diabetes mellitus	834 (24.8%)	44 (11.6%)	790 (26.4%)	<0.001
Current smoker	676 (20.1%)	34 (8.9%)	642 (21.5%)	<0.001
Dyslipidemia	1901 (56.5%)	163 (42.9%)	1738 (58.2%)	<0.001
Renal replacement therapy	38 (1.1%)	3 (0.8%)	35 (1.2%)	0.795
Dementia	28 (0.8%)	4 (1.1%)	24 (0.8%)	0.549
History of bleeding	322 (9.6%)	45 (11.8%)	277 (9.3%)	0.109
CHA2DS2-VASc score				<0.001
- 0	198 (5.9%)	4 (1.1%)	194 (6.5%)	
- 1	422 (12.5%)	21 (5.5%)	401 (13.4%)	
- ≥2	2747 (81.6%)	355 (93.4%)	2392 (80.1%)	
HAS-BLED score				<0.001
- 0	489 (14.5%)	25 (6.6%)	464 (15.5%)	
- 1–2	2354 (69.9%)	281 (73.9%)	2073 (69.4%)	
- ≥3	524 (15.6%)	74 (19.5%)	450 (15.1%)	
Antiplatelet	882 (26.2%)	81 (21.3%)	801 (26.8%)	0.022
Anticoagulant	2535 (75.3%)	293 (77.1%)	2242 (75.1%)	0.384
- Warfarin	2310 (68.6%)	276 (72.6%)	2034 (68.1%)	0.073
- NOACs	225 (6.7%)	17 (4.5%)	208 (7.0%)	0.067
For warfarin group				
- Time in therapeutic range (%)	53.5 ± 26.4	53.0 ± 26.2	53.6 ± 26.4	0.722
- Time under therapeutic range (%)	32.1 ± 27.6	29.9 ± 26.2	32.4 ± 27.8	0.158
- Time above therapeutic range (%)	14.1 ± 17.5	17.0 ± 19.9	13.7 ± 17.1	0.010
- Baseline INR	2.2 ± 0.8	2.4 ± 0.9	2.2 ± 0.8	<0.001

Data presented as mean ± standard deviation or number and percentage; A *p*-value < 0.05 indicates statistical significance. Abbreviations: BW, body weight; NVAF, non-valvular atrial fibrillation; CIED, cardiac implantable electronic device; TIA, transient ischemic attack; NOACs, non-vitamin K antagonist oral anticoagulants; INR, international normalized ratio

**Table 2:** Rates of clinical outcomes compared among different body weight groups.

	All (*n* = 3367)	BW < 50 (*n* = 380)	BW ≥ 50 (*n* = 2987)	*p*-Value
Ischemic stroke/TIA	99 (2.9%)	23 (6.1%)	76 (2.5%)	<0.001
Major bleeding	148 (4.4%)	27 (7.1%)	121 (4.1%)	0.006
ICH	48 (1.4%)	11 (2.9%)	37 (1.2%)	0.010
Death	260 (7.7%)	63 (16.6%)	197 (6.6%)	<0.001
Ischemic stroke/TIA or major bleeding or death	414 (12.3%)	90 (23.7%)	324 (10.8%)	<0.001

Data presented as number and percentage; A *p*-value < 0.05 indicates statistical significance. Abbreviations: BW, body weight; TIA, transient ischemic attack; ICH, intracerebral hemorrhage.

## Supplementary Materials

The following are available online at https://www.mdpi.com/2077-0383/9/9/2713/s1, Figure S1: Distribution of body weight among male patients (A), female patients (B), patients aged ≥ 65 years (C), and patients aged < 65 years (D); Figure S2: Restricted cubic splines graph showing Hazard ratio (95%CI) stratified by gender (A and B) and age group (C and D) for ischemic stroke/transient ischemic attack (TIA) (A, C) and major bleeding (B, D) in patients with different body weight adjusted for age, sex, and comorbid conditions (coronary artery disease, heart failure, smoking status, diabetes, hypercholesterolemia, hypertension, history of ischemic stroke, history of major bleeding, renal replacement therapy, cardiac implantable electronic devices). Cox regression models with restricted cubic splines was used for analysis.
