# Supplementary material for: Low Body Weight Increases the Risk of Ischemic Stroke and Major Bleeding in Atrial Fibrillation: The COOL-AF Registry
Source: J Clin Med. 2020 Aug 22;9(9):2713. doi: 10.3390/jcm9092713 (PMC7565915; doi:10.3390/jcm9092713)
Supplement: Supplementary file 1 [file jcm-09-02713-s001.pdf]

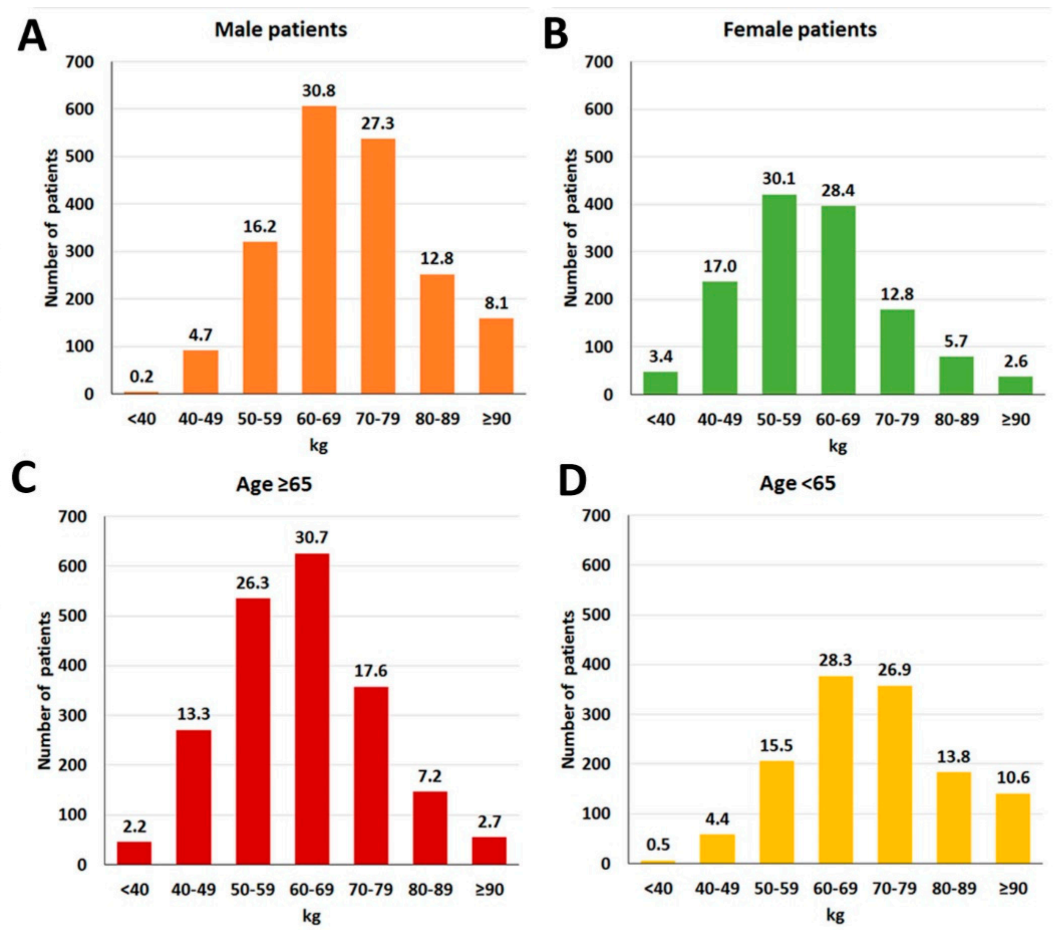

Figure S1. Distribution of body weight among male patients (**A**), female patients (**B**), patients aged ≥65 years (**C**), and patients aged <65 years (**D**).

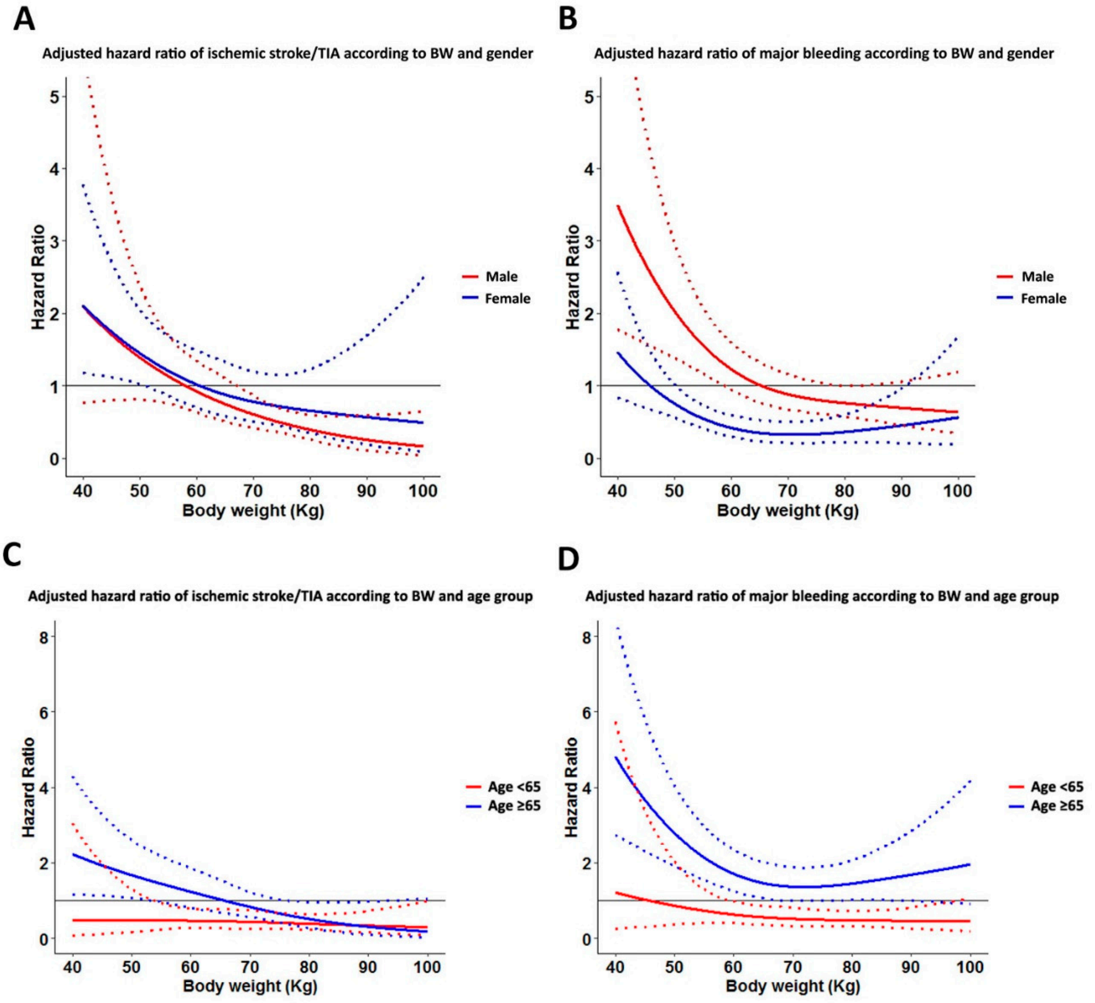

Figure S2. Restricted cubic splines graph showing Hazard ratio (95%CI) stratified by gender (**A** and **B**) and age group (**C** and **D**) for ischemic stroke/transient ischemic attack (TIA) (**A**, **C**) and major bleeding (**B**, **D**) in patients with different body weight adjusted for age, sex, and comorbid conditions (coronary artery disease, heart failure, smoking status, diabetes, hypercholesterolemia, hypertension, history of ischemic stroke, history of major bleeding, renal replacement therapy, cardiac implantable electronic devices). Cox regression models with restricted cubic splines was used for analysis.
